# Affective Working Memory in Depression

**DOI:** 10.1037/emo0001130

**Published:** 2022-11-28

**Authors:** Annabel Songco, Shivam D. Patel, Katy Dawes, Evangeline Rodrigues, Cliodhna O’Leary, Caitlin Hitchcock, Tim Dalgleish, Susanne Schweizer

**Affiliations:** 1 School of Psychology, University of New South Wales Sydney; 2 MRC Cognition and Brain Sciences Unit, University of Cambridge; 3 Melbourne School of Psychological Sciences, University of Melbourne; 4 Department of Psychology, University of Cambridge

**Keywords:** working memory capacity, emotion, depression, complex span, imagery

## Abstract

Depressed individuals show a wide range of difficulties in executive functioning (including working memory), which can be a significant burden on everyday mental processes. Theoretical models of depression have proposed these difficulties to be especially pronounced in affective contexts. However, evidence investigating affective working memory (WM) capacity in depressed individuals has shown mixed results. The preregistered study used a complex span task, which has been shown to be sensitive to difficulties with WM capacity in affective relative to neutral contexts in other clinical groups, to explore affective WM capacity in clinical depression. Affective WM capacity was compared between individuals with current depression (*n* = 24), individuals in remission from depression (*n* = 25), and healthy controls (*n* = 30). The results showed that, overall, WM capacity was more impaired in the context of negative distractor images, relative to neutral images. Furthermore, those with a lifetime history of depression (individuals with current depression and individuals remitted from depression), performed worse on the task, compared to healthy controls. However, there was no support for the greater disruption of WM capacity in affective compared to neutral contexts in those with a lifetime history of depression. These findings’ implications for current models of depression are discussed.

Cognitive difficulties are common in depressed individuals (Rock et al., 2014; Snyder, 2013), often persisting in patients remitted from depression (e.g., Hasselbalch et al., 2011). Theoretical frameworks exploring cognitive difficulties in depression have highlighted the role of working memory (WM) problems (Baddeley, 2003). WM is broadly defined here^1^ as a set of mental processes that make information temporarily available in capacity-limited storage for ongoing information processing, while inhibiting attention to distracting information (Trutti et al., 2021). Reduced WM capacity is proposed to contribute to the maintenance of depressive states by limiting the cognitive resources available to (a) engage in adaptive emotion regulatory processes (Joormann & Tanovic, 2015) and (b) override maladaptive cognitive biases (for reviews see: Everaert & Koster, 2020; LeMoult & Gotlib, 2019). WM capacity is required to successfully regulate emotions and override cognitive biases by maintaining situationally adaptive emotion regulatory goals and holding affective information in mind when it is helpful to an individual’s current goals, while ignoring affective information that distracts from these goals (Everaert & Koster, 2020; Schweizer et al., 2019). Importantly, it has been suggested that affective WM capacity (i.e., WM capacity applied in affective contexts) may be malleable and constitute a promising target for interventions (Jopling et al., 2020). The current study therefore examined affective WM capacity across depressive states.

We focus on affective WM capacity, as individuals with depression have been argued to experience difficulties specifically with the manipulation of negative information in WM (e.g., LeMoult & Gotlib, 2019). Difficulties with the removal of negative information from WM, what Joormann et al. (2011) termed “sticky thoughts,” have been proposed to propagate ruminative thinking in depression (Joormann & Tanovic, 2015). Rumination (i.e., repetitive negative thinking) is a maladaptive emotion regulation strategy that has been proposed to be central to both the etiology and maintenance of depression (Watkins & Roberts, 2020). Studies empirically investigating the impact of affective information on WM capacity have used complex span tasks (e.g., Hubbard, Hutchison, Hambrick et al., 2016), which are thought to reflect a natural representation of everyday difficulties experienced by individuals suffering from emotional disorders (Schweizer & Dalgleish, 2011, 2016).

Complex span tasks require individuals to simultaneously engage in two tasks competing for shared cognitive resources (Conway et al., 2005). One task is an operation task (e.g., mathematical operation, semantic processing), which engages cognitive capacity away from the second task component, the storage task. The storage task requires individuals to maintain memoranda (e.g., words, digits) in WM throughout the operation task(s). An example of a complex span task is the reading span task (Daneman & Carpenter, 1980). This task requires individuals to make a semantic judgment about a sentence (operation task), while maintaining an unrelated word presented at the end of the sentence in WM for later recall (storage task). Using a modified affective reading span task, we showed that the affective enhancement effect (i.e., improved cognitive performance in the presence of affectively salient information; Markovic et al., 2014) observed in healthy individuals was preserved in depressed individuals (Schweizer et al., 2018). Specifically, depressed individuals showed better memory for words following affective compared to neutral sentences. Other studies, however, have shown affective impairment effects (i.e., reduced performance in the presence of affectively salient stimuli) using similar variants of the reading span task in healthy (Garrison & Schmeichel, 2019) and dysphoric (Hubbard, Hutchison, Hambrick, et al., 2016; Hubbard, Hutchison, Turner, et al., 2016) individuals. Findings on WM capacity in depression then are mixed.

Beyond WM capacity, simple WM storage (i.e., without concomitant information processing demands) and WM updating has been studied in individuals with depressive symptoms using a range of simple span tasks and updating tasks, respectively. These studies have typically failed to show differential effects of affective relative to neutral stimuli on WM accuracy in depression, some have shown effects on reaction time (RT; Berman et al., 2011; Bertocci et al., 2012; Foland-Ross et al., 2013; Joormann et al., 2011; Tavitian et al., 2014; Yoon et al., 2014). These processing speeds deficits arguably reflect difficulties disengaging from affective information (impaired disengagement hypothesis; Koster et al., 2011). This difficulty with affective disengagement in those with depression does not appear to be limited to WM. For example, the impact of negative stimuli on inhibition performance on the affective GoNogo has been shown to increase as a function of depressive symptoms (Price et al., 2021). Theoretical models and some empirical evidence then point to depressed individuals having difficulties with updating affective information in WM. To understand affective WM capacity in depression better, the current study conducted a preregistered investigation of affective WM capacity in individuals with depression, in remission from depression, and healthy controls using a complex span task including a perceptual operation component that has been shown to be sensitive in clinical groups (Schweizer & Dalgleish, 2016).

## The Present Study

The present study used the Affective Picture Span Paradigm (APSP) to measure affective WM capacity (Figure S1). The APSP has been shown to reliably lead to a reduction in WM capacity for negative relative to neutral distractors in healthy individuals and clinical groups, (Schweizer & Dalgleish, 2016). We predicted that (H1a) all participants would remember fewer words when presented in the context of negative distractor images; (H1b) individuals with a lifetime history of depression would recall fewer words overall compared to never-depressed controls; (H1c) this would be seen especially in the negative relative to the neutral ASPS condition. And these effects would be strongest in currently depressed individuals H2.

## Method

### Participants

Eighty participants (*M* age = 38.99, *SD* = 14.97; 63% females; Table S1 for participant characteristics) were recruited from the University of Cambridge, United Kingdom, as well as via community advertisements (see protocol and updates for sample size justification: https://osf.io/e4yas/). For inclusion and exclusion details see the supplementary results in the online supplemental materials. The final sample included individuals with current MDD (*N* = 24), remitted MDD (*N* = 25), and never-depressed controls (*N* = 30).

### Measures

#### Working Memory

Affective WM capacity was measured with the APSP (Schweizer & Dalgleish, 2016; see online supplemental materials for details). The APSP comprised two components, a target storage task and an operation task, which were performed simultaneously in the presence of either neutral or negative background images (for a full list of the images included see Table S2). For the operation task, participants counted shapes (4–6 shapes per trial) that appeared before and after a word was presented. The proportion of words recalled correctly were computed for each trial size (4–7 items) and valence (neutral, negative).

#### Depression

Depressive symptoms were assessed using the Beck Depression Inventory (BDI-II; Beck et al., 1996), a well-validated measure of symptoms of depression.

#### Anxiety

The well-validated State–Trait Anxiety Inventory (Spielberger et al., 1983) was used to assess state and trait anxiety (STAI-S/T).

#### Verbal IQ

The National Adult Reading test (NART; Nelson, 1982) was used to assess whether group differences in WM capacity were due to estimated premorbid verbal intelligence.

### Procedure

Ethical approval was obtained from the NHS Research Ethics Committee, East of England (11/H0305/1). Participants completed the questionnaires and NART, before the APSP. The presentation of the APSP neutral and negative conditions was counterbalanced. Participants were compensated for their time (£6 per hour). See the online supplemental materials for our data analysis description.

### Transparency and Openness

**Figure 1 fig1:**
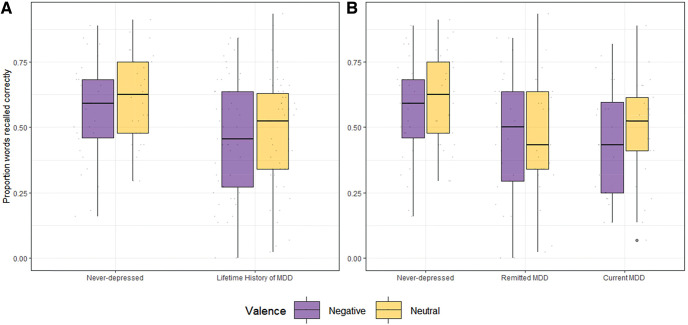
Proportion Recall Across Depressive States and Valence *Note*. Panel A shows the proportions of words recalled correctly in the negative (purple/dark gray) and neutral (yellow/light gray) conditions of the Affective Picture Span Paradigm (Schweizer & Dalgleish, 2016), by never-depressed individuals (left) and those with a lifetime history of major depressive disorder (MDD) (right). Individual data points are represented as well as the median score, thick black line in boxplot. The thin black bars represent the standard deviation within each group and condition. Panel B illustrates words recalled correctly in the negative (purple/dark grey) and neutral (yellow/light grey) conditions, by never-depressed individuals (left), individuals remitted from MDD (middle) and currently depressed individuals (right). Depressive status was determined by the Semi-structured Clinical Interview for *DSM–V* Disorders (First et al., 2015). The task includes pictures from the International Affective Picture System IAPS database. See the online article for the color version of this figure.

We report how we determined our sample size, all data exclusions, all manipulations, and all measures in the preregistered (https://osf.io/escdr) study. All frequentist analyses were run using SPSS Version 26 (IBM Corp., 2017), Figure 1 was created in R (R Core Team, 2013) with ggplot2 and cowplot. JASP (JASP Team, 2021) was used for Bayesian analyses. The data has not been made open access due to the restrictions on the ethical approval of the study.[Fig fig1]

## Results

All means and standard deviations across depressive states and valence are reported in Table 1.[Table tbl1]

In line with H1a, there was a moderate, significant effect of valence (*F*[1, 77] = 5.59, *p* = .021, η_p_^2^ = .07, BF_10_ = 1.28) on WM capacity, with performance being more impaired in the context of negative images relative to neutral images (Figure 1A). Supporting H1b, there was a moderate, significant effect of depression (*F*[1, 77] = 6.68, *p* = .012, η_p_^2^ = .08, BF_10_ = 3.93; Figure 1B). That is, WM capacity in participants with a lifetime history of depression was lower compared to never-depressed participants. However, contrary to H1c, there was no significant interaction (*F*[1, 77] = 1.51, *p* = .222, η_p_^2^ = .02, BF_10_ = 2.09).

Partially, confirming H2 there was a moderate, significant effect of group (*F*[2, 76] = 3.30, *p* = .042, η_p_^2^ = .08; BF_10_ = 1.91; Table 1). Pairwise comparisons showed that never-depressed controls performed significantly better on the APSP vs. participants with current MDD (*F*[1, 52] = 5.93, *p* = .018, η_p_^2^ = .10; BF_10_ = 20.45) and those remitted from MDD (*F*[1, 53] = 4.58, *p* = .037, η_p_^2^ = .08; BF_10_ = 8.69). Performance did not significantly differ between participants with current MDD and those remitted from MDD (*F*[1, 47] < 1, *p* = .942, η_p_^2^ = .02; BF_10_ = .21). Finally, contrary to H2, there was no significant interaction (*F*[2, 76] = 1.14, *p* = .324, η_p_^2^ = .03; BF_10_ = .57; Table 1). Exploratory analyses showed that this was not due to a trade-off with accuracy on the operation task (see supplemental results in the online supplemental materials).

## Discussion

The present preregistered study investigated the impact of affective information on WM capacity in depressed individuals. The results demonstrated that affective distraction on the APSP introduced through task-irrelevant background images reliably reduced WM capacity compared to neutral background images. That is, storage performance was reduced arguably due to attention being allocated to the distracting images. This is noteworthy as our meta-analysis indicated that performance on WM tasks in affective, compared to neutral, contexts (i.e., including affective and neutral stimuli, respectively) is not impacted in mentally well individuals (Schweizer et al., 2019). The dual-task demands of our complex span task (which involved visuospatial search and word maintenance) completed in the context of task-irrelevant affective versus neutral distraction provides an ecologically valid analogue of completing higher order mental processes while confronted with goal-irrelevant affective information (e.g., feelings, thoughts, or memories).

In contrast with our predictions, depression history (lifetime vs. never depressed) and depression state (never, remitted, current) did not appear to interact with the impact of these affective distractors. That is, while WM capacity was lower in individuals with a lifetime history of depression compared to never-depressed individuals, the difference in performance was not greater in the affective compared to the neutral condition. In fact, the results from the Bayesian analyses provided some support for WM capacity in never-depressed individuals being more impaired by the affective condition compared to those with a lifetime history of MDD.

These findings are in contrast with studies that have found valence-specific reductions in WM capacity, using variants of the reading span task with affective information (Hubbard, Hutchison, Hambrick, et al., 2016; Hubbard, Hutchison, Turner, et al., 2016). Moreover, meta-analytic evidence showing moderate to large differences between healthy individuals and those with symptoms of depression in attention maintenance toward negative images (Suslow et al., 2020) suggests that sustained processing of the aversive background images should interfere with WM encoding and maintenance, as well as the operation task in individuals with depression. However, as for WM capacity, there was no evidence of greater impairment on the shape detection operational task in individuals with a lifetime history of depression compared to those who had never been depressed.

A potential account for these findings is the included stimuli’s limited impact. That is, the negative stimuli included in the current study, generic aversive images, may not have triggered deep self-referential processing that would take away cognitive resources from the goal-relevant task components in individuals with a history of depression. In support of this account, studies that facilitated depth of processing using affective version of the reading span task showed reduced recall of unrelated words on these negative trials compared to healthy individuals (Hubbard, Hutchison, Hambrick, et al., 2016; Hubbard, Hutchison, Turner, et al., 2016). However, using different version of a depressogenic reading span task (discussed above), which similarly engaged self-referential processing, we showed no interaction effect between depressive status and valence (Schweizer et al., 2018). Together, these findings suggest that limited affective salience may not, or may only partially, account for the lack of an interaction between depression status and valence.

An alternative account for the absence of affective impairment in individuals with a lifetime history of depression compared to never-depressed individuals is the high cognitive load imposed by the APSP. The task is made cognitively demanding by simultaneously engaging participants in two mentally challenging tasks. The task demands may have been sufficient to reduce content processing prohibiting any depressogenic rumination about the content of the affective images. Research on anxiety has previously shown that high cognitive load can reduce or even eliminate the effects of fear conditioning (Yates et al., 2010) and encoding of highly aversive (trauma-analogue) information (Holmes et al., 2010). Similarly, Hu et al. (2021) showed that individuals with subthreshold depression showed greater directed forgetting of negative information under conditions of high load. That is, high cognitive load may have constrained more elaborate processing of the negative stimulus material. Together, these findings suggest that under high cognitive load individuals with depression appear able to inhibit distraction from goal-irrelevant affective information. Finally, differences in task design (e.g., visuospatial vs. verbal complex span task) and study samples (e.g., symptoms severity) may contribute to mixed effects, when exploring subtle impacts of affective information on WM. Different assessment modalities may be more sensitive to group difference. In the absence of behavioral differences in comparison with never-depressed controls, individuals remitted from MDD showed differential recruitment of cognitive control network in response to negative and positive distractors relative to healthy individuals (Kerestes et al., 2012). Future research exploring the nature of affective WM capacity in depression, then, may benefit from the inclusion of multimodal outcomes.

The current study constitutes a significant addition to the discourse about the nature of affective WM capacity in individuals with depression. The study was preregistered and adequately powered to detect an interaction effect based on work using this task in another clinical sample. In line with a growing number of studies (e.g., Joormann et al., 2011), the results show no greater impairment in WM performance in affective compared to neutral contexts in depressed individuals. Our findings contribute to accumulating evidence that theoretical models on the role of WM in depression may need to consider that WM problems in depression are only minimally moderated by valence.

## Supplementary Material

10.1037/emo0001130.supp

## Figures and Tables

**Table 1 tbl1:** WM Performance Across Depressive State and Valence

	Hypothesis 1 (a–c)			Hypothesis 2 (a–b)^a^			
	Never-depressed	Lifetime history MDD		Never-depressed	Remitted MDD	Current MDD	
Measures	(*n* = 30)	(*n* = 49)	Total	(*n* = 30)	(*n* = 25)	(*n* = 24)	Total
Neutral *M* (*SD*)	.61 (.17)	.48 (.21)	.53 (.21)	.61 (.17)	.47 (.23)	.48 (.20)	.53 (.21)
Negative *M* (*SD*)	.56 (.19)	.46 (.23)	.50 (.22)	.56 (.19)	.47 (.25)	.45 (.21)	.50 (.22)
Overall WM *M* (*SE*)	.59 (.04)	.47 (.03)		.59 (.04)	.47 (.04)	.47 (.04)	
Effect size (η_p_^2^)							
Valence	.07			.05			
Group	.08			.08			
Valence × Group	.02			.03			
*Note*. Neutral = proportion of words recalled correctly in the context of neutral images; Negative = proportion of words recalled correctly in the context of negative images; Overall WM = overall working memory; Never-depressed = individuals with no history of MDD; Lifetime history MDD = combined Current MDD and Remitted MDD groups; Total = proportion of words recalled correctly averaged across neutral and negative trials; Valence = main effect of valence comparing proportion of negative to proportion of neutral words recalled; Group = main effect of group for hypothesis 1a-c this compares never-depressed individuals to individuals with a lifetime history of depression and for hypothesis 2a–b individuals performance is compared across the never-depressed, remitted, and currently depressed groups; Valence x Group = interaction effect between valence and group.							
^a^ We additionally, as per our preregistration, explored the effect of valence and depression status on words recalled in the correct position. The proportion of neutral words recalled in the correct position was, never-depressed: *M* = .46, *SD* = .20; remitted-recurrent MDD: *M* = .33, *SD* = .24 and current MDD: *M* = .34, *SD* = .21. The proportion of negative words recalled in the correct position was, never-depressed: *M* = .40, *SD* = .20; remitted-recurrent MDD: *M* = .32, *SD* = .26 and current MDD: *M* = .28, *SD* = .20. Repeating all analyses with the proportion words recalled in the correct position yielded the same pattern of results, showing a significant effect of valence (*F*(1, 77) = 7.47, *p* = .008, η_p_^2^ = .09; BF_10_ = 3.33) but no interaction of valence with lifetime history of depression (*F*(1, 77) < 1, *p* = .502, η_p_^2^ = .01; BF_10_ = .70) or current depression status (Never, Remitted, Current), (*F*(2, 76) < 1, *p* = .419, η_p_^2^ = .02; BF_10_ = .27).							

## Footnotes

1 It should be noted there is no universally agreed definition of WM (Cowan, 2017).
