# Supplementary material for: Affective Working Memory in Depression
Source: Emotion. 2022 Nov 28;23(6):1802–7. doi: 10.1037/emo0001130 (PMC10448741; doi:10.1037/emo0001130)
Supplement: Supplementary file 1 [file EMO-2021-2868_Supplementary_Materials.docx]

**Supplementary Materials**

**AFFECTIVE WORKING MEMORY IN DEPRESSION**

Annabel Songco, Shivam D. Patel, Evangeline Rodrigues, Cliodhna O’Leary, Caitlin Hitchcock, Tim Dalgleish and Susanne Schweizer

**Author note:**

This work was funded by the UK Medical Research Council (Grant Reference: SUAG/043 G101400) awarded to Tim Dalgleish. The research was partly supported by the National Institute for Health Research Cambridge Biomedical Research Centre. Susanne Schweizer was funded by a Wellcome Trust fellowship (209127/Z/17/Z), the National Institute of Mental Health (R61-MH121560), National Health and Medical Research Council (APP1184136) and Jacobs Foundation (Young Scholar Award). Caitlin Hitchcock was funded by the Economic and Social Research Council (ES/R010781/1). The funding sources did not have any involvement in the study design; in the collection, analysis and interpretation of data; in the writing of the report; and in the decision to submit the article for publication. For the purpose of open access, the author has applied a CC BY public copyright licence to any Author Accepted manuscript version arising from this submission.

*Author contributions:* SS and TD designed the study. SDP, KD, ER, COL collected the data. CH was the clinical database manager. AS, SS and TD analysed the data. SS and AS wrote the manuscript. All authors commented on the manuscript.

*Acknowledgements:* The authors would like to thank all participants who helped with this research.

**Supplementary introduction**

**Figure S1**

*Affective Picture Span Paradigm Sample Trial*
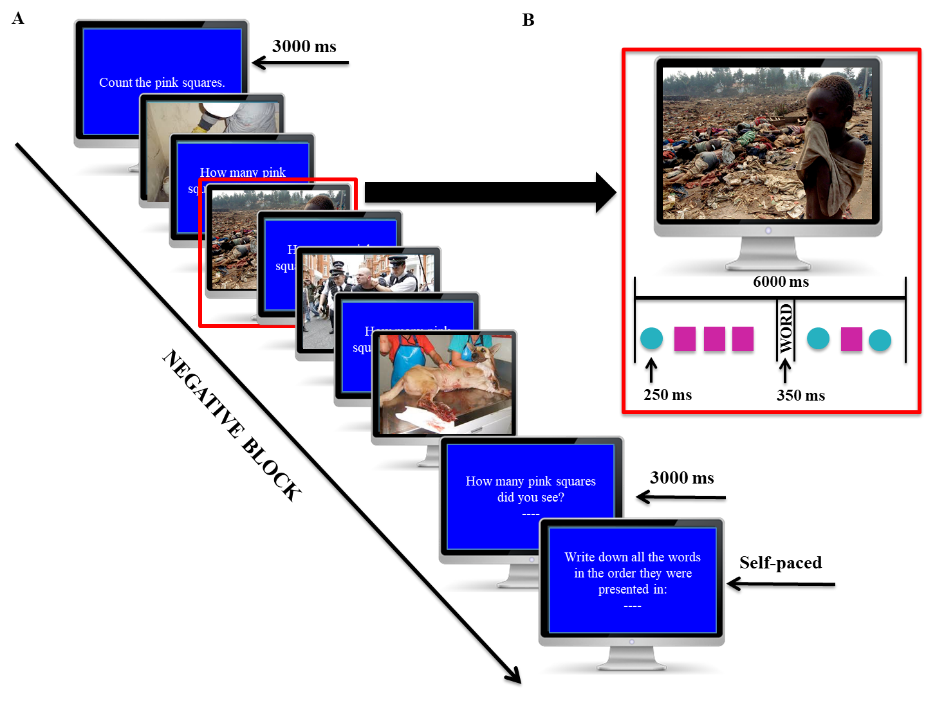


*Note.* Panel (A) shows a negatively valenced trial block comprising 4 trials. Each trial includes two slides. On the first slide shapes and a word appear (see panel B for details) over a background image (presented for 6s). After each image and word pairing a second screen appears for 3s. On this second screen participants indicate how many target shapes they saw in this trial. In this example, the target shapes are the pink squares. That is, participants have to count the pink squares that appear during the trial, while ignoring the turquoise circles. At the end of each block (4-7 trials) a slide appears (final slide in panel A), which instructs participants to report all words they have seen in the current block, in the order in which they appeared. Panel (B) illustrates the time course of a single trial in which four target shapes are presented. Each shape is presented for 250ms. The word, which appears halfway through the trial (at 3s), is presented for 350ms.

**Supplementary methods**

**Participant recruitment and inclusion/exclusion**

Participants were recruited from across a range of depressive scores on the BDI-II (Beck et al., 1996). Data from one currently depressed individual had to be excluded due to the task malfunctioning, leaving a total sample of 79 participants. Participants met study inclusion if they were above 18 years old, fluent in English, had no history of head injury or current neurological disorders, and had normal or corrected hearing and vision. Depressive status and history was determined using the Structured Clinical Interview for DSM Disorders (First et al., 2015). The major depressive disorder (MDD) diagnosis could not be part of a bipolar disorder nor due to bereavement.

As indicated in the pre-registration, the sample size (per-registered *N* = 60; see <https://osf.io/e4yas/> for justification for deviation from pre-registered protocol and updated sample size justification) was determined to have 90% power to detect a group (current MDD, remitted MDD, never-depressed) by valence (negative, neutral) interaction as reported in our previous study (Schweizer & Dalgleish, 2016; η_p_^2^ = 0.19). Groups did not differ in age (*F*(2, 76) = 1.33, *p* = .270; η_p_^2^ = 0.03), gender (χ²(4)) = 4.80, *p* = .309), verbal IQ (*F*(2, 76) = 1.50, *p* = .229; η_p_^2^ = 0.04). In addition, there were no significant differences between groups in education, ethnicity or household income (all *ps* >.05). As expected, there were significant differences between groups in depressive symptomology (*F*(2, 76) = 60.13, *p* = < .001; η_p_^2^ = 0.61) as well as state (*F*(2, 76) = 15.35, *p* <.005; η_p_^2^ = 0.29) and trait (*F*(2, 76) = 29.48, *p* <.001; η_p_^2^ = 0.44) anxiety.

**Affective Picture Span Paradigm Details**

Affective WM capacity was measured with the AASP (Schweizer & Dalgleish, 2016). The APSP comprised two components, a target storage task and an operation task, which were performed simultaneously in the presence of either neutral or negative background images. For the target storage task, participants were required to retain a set of 4-7 words (trial size), presented one word at a time against a background image. Each trial size was presented twice for each valence condition (neutral, negative), resulting in 16 blocks. The valence of the APSP was manipulated by presenting emotionally negative or neutral background images, which were selected from the International Affective Picture System (for a full list of the images included see Table S2). For the operation task, participants counted shapes (4-6 shapes per trial) that appeared before and after a word was presented. Participants were prompted to accurately enter the number of target shapes that appeared after each trial using the computer keyboard (e.g., ‘How many pink squares did you see?’). At the end of each block, participants were instructed to recall as many words as they could remember. The proportion of words recalled correctly were computed for each trial size (4 -7 items) and valence (neutral, negative), then proportions were calculated for the neutral and depressed conditions across all trials, irrespective of whether words were recalled in the correct position.

**Data analysis**

To investigate the first hypothesis (H1a-c), a group (lifetime history of MDD, never-depressed) by valence (neutral, negative) repeated measures mixed-model ANOVA was conducted with proportion words recalled correctly as outcome variable. Valence was entered as the within-subjects factor and group entered as the between-subjects factor. The lifetime history of MDD group was derived from a combination of individuals with current MDD and remitted-recurrent MDD.

The above analysis was repeated to investigate the second hypothesis (H2) with group (current MDD, remitted-recurrent MDD, never-depressed) as the between-subjects factor and valence (neutral, negative) as the within-subjects factor. All assumptions for the models were met.

For each analysis inferences were made based on one-tailed (due to directional hypotheses) *p*-values with significance set at *p* < .05 as well as Bayes factors. Specifically, we report BF_10_, which quantifies the evidence in favour of the alternative hypothesis (compared to the null hypothesis). Where the conclusions from standard hypothesis testing and Bayesian inferences differ, this will be discussed.

**Table S1**

*Participant Characteristics*

|  | Never-depressed | | | Remitted-recurrent MDD | | | Current MDD | | |
| --- | --- | --- | --- | --- | --- | --- | --- | --- | --- |
|  | *n / M* | % / *SD* | Range | *n / M* | % / *SD* | Range | *n / M* | % / *SD* |  |
| Demographic characteristics |  |  |  |  |  |  |  |  |  |
| Age | 36.10 | 15.88 | 19-65 | 42.68 | 14.41 | 21-71 | 38.76 | 13.20 | 21-73 |
| Gender  Female  Male  Other | 19  11  -- | 63.33  36.67 |  | 13  10  1 | 54.17  41.67  4.17 |  | 19  6  -- | 76.00  24.00 |  |
| Education  GCSE  A-level  University  Postgraduate  Other | 4  11  10  5  -- | 13.33  36.67  33.33  16.67  -- |  | 2  8  6  7  1 | 8.33  33.33  25.00  29.17  4.17 |  | 2  10  7  6  -- | 8.00  40.00  28.00  24.00  -- |  |
| Verbal IQ | 104.59 | 7.71 | 82.09-116.05 | 106.52 | 6.61 | 88.62-116.05 | 102.82 | 7.88 | 88.62-119.96 |
| Household income | 48726.79 | 78025.31 | 0-300000 | 35020.79 | 31121.28 | 0-100000 | 40652.52 | 69226.73 | 0-331000 |
| Race^a^  Asian  Black  Mixed  White | 3  1  --  26 | 10.00  3.33  --  86.67 |  | --  --  1  23 | --  --  4.00  96.00 |  | 1  --  1  22 | 4.00  --  4.00  88.00 |  |
| Clinical characteristics |  |  |  |  |  |  |  |  |  |
| Depressive symptoms  Minimal (0-13)  Mild (14-19)  Moderate (20-28)  Severe (29-63) | 4.53  29  1  --  -- | 4.17  96.67  3.33  --  -- | 0-18 | 19.00  10  4  2  8 | 13.72  41.67  16.67  8.33  33.33 | 0-47 | 35.52  1  --  5  19 | 11.97  4.00  --  20.00  76.00 | 10-61 |
| State anxiety | 34.63 | 15.01 | 21-79 | 44.17 | 14.84 | 20-74 | 57.00 | 14.76 | 31-80 |
| Trait anxiety | 37.83 | 12.99 | 21-79 | 49.71 | 14.41 | 25-71 | 64.64 | 11.11 | 38-79 |

*Note. GCSE = Completed General Certificate of Secondary Education (refers to an academic qualification in a particular subject taken between the 9^th^ and 11^th^ year of core education in the UK); A-level = Completed Advanced level(s) is a subject-based school leaving qualification offered in the UK; University = Graduated from a university course; Postgraduate = Achieved postgraduate qualifications; Verbal IQ = Verbal intelligence quotient converted from the total number of errors on the National Adult Reading Test* (Nelson, 1982)*; Depressive symptoms = measured on the Beck Depression Inventory Version 2* (BDI-II; Beck et al., 1996)*; Minimal = BDI-II cut off for minimal depressive symptoms; Mild = BDI-II cut off for mild depressive symptoms; Moderate = BDI-II cut off for moderate depressive symptoms; Severe = BDI-II cut off for severe depressive symptoms; State anxiety = Symptoms of anxiety on the state-version of the Spielberger State-Trait Anxiety Inventory* (Spielberger et al., 1983)*; Trait anxiety = Trait levels of anxiety on the trait-version of the Spielberger State-Trait Anxiety Inventory* (Spielberger et al., 1983)*.*

**Table S1**

*International Affective Picture System Images Included in the Affective Picture Span Paradigm*

| Image Name | Image Number | Mean Valence | SD Valence | Mean Arousal | SD Arousal |
| --- | --- | --- | --- | --- | --- |
| **Negative** | | | | | |
|  | | | | | |
| Grieving Fem | 2141 | 2.44 | 1.64 | 5.00 | 2.03 |
| Hospital | 2205 | 1.95 | 1.58 | 4.53 | 2.23 |
| Bloody Kiss | 2352 | 2.09 | 1.50 | 6.25 | 2.10 |
| Baby | 2661 | 3.90 | 2.49 | 5.76 | 2.13 |
| Burn Victim | 3100 | 1.60 | 1.07 | 6.49 | 2.23 |
| Dying Man | 3230 | 2.02 | 1.30 | 5.41 | 2.21 |
| Injured Child | 3301 | 1.80 | 1.28 | 5.21 | 2.26 |
| Attack | 3500 | 2.21 | 1.34 | 6.99 | 2.19 |
| Soldier | 6212 | 2.19 | 1.49 | 6.01 | 2.44 |
| Aimed Gun | 6230 | 2.37 | 1.57 | 7.35 | 2.01 |
| Attack | 6313 | 1.98 | 1.38 | 6.94 | 2.23 |
| Attack | 6370 | 2.70 | 1.52 | 6.44 | 2.19 |
| Attack | 6510 | 2.46 | 1.58 | 6.96 | 2.09 |
| Attack | 6560 | 2.16 | 1.41 | 6.53 | 2.42 |
| Suicide | 6570 | 2.19 | 1.72 | 6.24 | 2.16 |
| Guns | 6830 | 2.82 | 1.81 | 6.21 | 2.23 |
| Police | 6831 | 2.59 | 1.50 | 5.55 | 2.16 |
| Police | 6834 | 2.91 | 1.73 | 6.28 | 1.90 |
| Police | 6838 | 2.45 | 1.44 | 5.80 | 2.09 |
| Meat Slicer | 7361 | 3.10 | 1.73 | 5.09 | 2.48 |
| Boxer | 8230 | 2.95 | 1.88 | 5.91 | 2.15 |
| Cemetery | 9000 | 2.55 | 1.55 | 4.06 | 2.25 |
| Needles | 9007 | 2.49 | 1.41 | 5.03 | 2.32 |
| Starving Child | 9040 | 1.67 | 1.07 | 5.82 | 2.15 |
| Stick Thru Lip | 9042 | 3.15 | 1.89 | 5.78 | 2.48 |
| Cows | 9140 | 2.19 | 1.37 | 5.38 | 2.19 |
| Dead Cows | 9181 | 2.26 | 1.85 | 5.39 | 2.41 |
| War Victim | 9250 | 2.57 | 1.39 | 6.60 | 1.87 |
| Dead Body | 9252 | 1.98 | 1.59 | 6.64 | 2.33 |
| Mutilation | 9253 | 2.00 | 1.19 | 5.53 | 2.40 |
| Hung Man | 9265 | 2.60 | 1.52 | 4.34 | 2.09 |
| Dirty | 9300 | 2.26 | 1.76 | 6.00 | 2.41 |
| Soldier | 9400 | 2.50 | 1.61 | 5.99 | 2.15 |
| Soldier | 9410 | 1.51 | 1.15 | 7.07 | 2.81 |
| Soldier | 9420 | 2.31 | 1.59 | 5.69 | 2.28 |
| Dead Man | 9433 | 1.84 | 1.19 | 5.89 | 2.60 |
| Corpse | 9490 | 3.60 | 1.72 | 5.57 | 2.13 |
| Porpoises | 9500 | 2.42 | 1.73 | 5.82 | 2.29 |
| Sick Kitty | 9561 | 2.68 | 1.92 | 4.79 | 2.29 |
| Dog | 9570 | 1.68 | 1.23 | 6.14 | 2.31 |
| Cat | 9571 | 1.96 | 1.50 | 5.64 | 2.50 |
| Skinhead | 9800 | 2.04 | 1.57 | 6.05 | 2.71 |
| Car Accident | 9920 | 2.50 | 1.52 | 5.76 | 1.96 |
| Fire | 9921 | 2.04 | 1.47 | 6.52 | 1.94 |
| Average rating |  | 2.36 | 0.50 | 5.87 | 0.73 |
| **Neutral** | | | | | |
|  | | | | | |
| Gannet | 1450 | 6.37 | 1.62 | 2.83 | 1.87 |
| Giraffes | 1601 | 6.86 | 1.51 | 3.92 | 2.07 |
| Butterfly | 1602 | 6.50 | 1.64 | 3.43 | 1.96 |
| Springbok | 1620 | 7.37 | 1.56 | 3.54 | 2.34 |
| Jaguar | 1650 | 6.65 | 2.25 | 6.23 | 1.99 |
| Neu Woman | 2038 | 5.09 | 1.35 | 2.94 | 1.93 |
| Baby | 2058 | 7.91 | 1.26 | 5.09 | 2.48 |
| Neutral Man | 2102 | 5.16 | 0.96 | 3.03 | 1.87 |
| Neutral Man | 2214 | 5.01 | 1.12 | 3.46 | 1.97 |
| Boys Reading | 2222 | 7.11 | 1.54 | 4.08 | 2.15 |
| Family | 2358 | 6.56 | 1.70 | 3.73 | 2.04 |
| Musician | 2487 | 5.20 | 1.80 | 4.05 | 1.92 |
| Man | 2500 | 6.16 | 1.54 | 3.61 | 1.91 |
| Woman | 2514 | 5.19 | 1.09 | 3.50 | 1.81 |
| Harvest | 2515 | 6.09 | 1.54 | 3.80 | 2.12 |
| Quilting | 2518 | 5.67 | 1.66 | 3.31 | 1.88 |
| Picnic | 2560 | 6.34 | 1.53 | 3.49 | 2.07 |
| Bakers | 2579 | 5.53 | 1.35 | 3.85 | 2.00 |
| Family | 2598 | 7.19 | 1.30 | 3.73 | 1.84 |
| Smoking | 2749 | 5.04 | 1.39 | 3.76 | 2.03 |
| Balloons | 2791 | 6.64 | 1.70 | 3.83 | 2.09 |
| Flower | 5030 | 6.51 | 1.73 | 2.74 | 2.13 |
| Nature | 5201 | 7.06 | 1.71 | 3.83 | 2.49 |
| Galaxy | 5300 | 6.91 | 1.80 | 4.36 | 2.62 |
| Boat | 5395 | 5.34 | 1.21 | 4.23 | 2.03 |
| Satellite | 5471 | 5.21 | 1.18 | 3.26 | 2.05 |
| Mushrooms | 5532 | 5.19 | 1.69 | 3.79 | 2.20 |
| Winter Street | 5635 | 6.25 | 1.56 | 3.97 | 2.03 |
| Mountains | 5660 | 7.27 | 1.59 | 5.07 | 2.62 |
| Cave | 5661 | 5.96 | 1.41 | 4.15 | 2.30 |
| Field | 5711 | 6.62 | 1.65 | 3.03 | 1.96 |
| Plant | 5740 | 5.07 | 1.27 | 2.36 | 1.77 |
| Nature | 5760 | 8.05 | 1.23 | 3.22 | 2.39 |
| Field | 5764 | 6.74 | 1.64 | 3.55 | 2.32 |
| Courtyard | 5779 | 7.33 | 1.42 | 3.57 | 2.30 |
| Beach | 5836 | 7.25 | 1.39 | 4.28 | 2.41 |
| Lightning | 5950 | 5.99 | 2.07 | 6.79 | 1.98 |
| Skyline | 5994 | 6.80 | 1.75 | 4.61 | 2.31 |
| Truck | 7130 | 4.77 | 1.03 | 3.35 | 1.90 |
| Rug | 7179 | 5.06 | 1.05 | 2.88 | 1.97 |
| Beads | 7207 | 5.15 | 1.46 | 3.57 | 2.25 |
| Plate | 7233 | 5.09 | 1.46 | 2.77 | 1.92 |
| Desserts | 7320 | 6.54 | 1.63 | 4.44 | 2.12 |
| Golfer | 8311 | 5.88 | 1.67 | 3.57 | 2.35 |
| Average rating |  | 6.17 | 0.89 | 3.79 | 0.84 |

**Supplementary results**

**Operation task performance.** To explore (these analyses were not pre-registered) whether the above effects may be accounted for by accuracy on the storage versus operation task in the APSP, the group analyses was repeated with accuracy on the operation task as the outcome. There was a significant, moderate difference in accuracy across valence conditions (*F*(1, 76) = 7.72, *p* = .007, η_p_^2^ = .09; BF_10_ = 18.87), with higher accuracy in the neutral condition (*M* = .69, *SD* = .20) relative to the negative condition (*M* = .65, *SD* = .20). However, there was no significant difference in accuracy on the operation task across the three groups (*F*(2, 76) = .699, *p* = .500, η_p_^2^ = .02; BF_10_ = 0.16). There was also no significant interaction of *valence x group* (*F*(2, 76) = .268, *p* = .765, η_p_^2^ = .01; BF_10_ = 0.40). That is, never-depressed participants (neutral valence *M* = .72, *SD* = .18; negative valence *M* = .67, *SD* = .19; overall accuracy 69%), participants remitted from MDD (neutral valence *M* = .69, *SD* = .20; negative valence *M* = .66, *SD* = .22; overall accuracy = 68%), and current MDD participants (neutral valence *M* = .65, *SD* = .22; negative valence *M* = .61, *SD* = 20; overall accuracy = 63%) did not differ in accuracy across valence conditions.
